# Effects of resistance training in healthy older people with sarcopenia: a systematic review and meta-analysis of randomized controlled trials

**DOI:** 10.1186/s11556-021-00277-7

**Published:** 2021-11-11

**Authors:** Nan Chen, Xiangfeng He, Yuwei Feng, Barbara E. Ainsworth, Yu Liu

**Affiliations:** 1 Key Laboratory of Exercise and Health Sciences of Ministry of Education, Shanghai University of Sport, Shanghai, China; 2 Department of Rehabilitation, Xinhua Hospital Chongming Branch, Shanghai Jiao Tong University, Shanghai, China; 3 Department of Rehabilitation, Xinhua Hospital, Shanghai Jiao Tong University, Shanghai, China; 4 College of Health Solutions, Arizona State University, Phoenix, AZ USA

**Keywords:** Sarcopenia, Resistance training, Body composition, Muscle strength, Muscle performance

## Abstract

**Objective:**

We conducted a meta-analysis to analyze the effects of resistance training on measures of body composition, muscle strength, and muscle performance in older people with sarcopenia.

**Methods:**

All randomized controlled trials on the effects of resistance training on outcome variables in older people with sarcopenia were searched on Pubmed, Embase, Cochrane Library, the China National Knowledge Infrastructure (CNKI), and Wanfang. Data from January 2010 to October 2020 were reviewed. Two researchers extracted data and evaluated the quality of the studies that met the inclusion criteria independently. Meta-analysis for pre-post changes were calculated as standardized mean difference (SMD) with 95% confidence intervals (CI).

**Results:**

Fourteen studies meeting inclusion criteria included 561 healthy older adults (age 65.8 to 82.8) with sarcopenia. Compared with the control group, resistance training had positive effects on body fat mass (SMD = -0.53, 95% CI − 0.81 to − 0.25, *p* = 0.0002, *I*^*2*^ = 0%), handgrip strength (SMD = 0.81, 95%CI 0.35 to 1.27, *p* = 0.0005, *I*^*2*^ = 81%), knee extension strength (SMD = 1.26, 95% CI 0.72 to 1.80, *p* < 0.0001, *I*^*2*^ = 67%), gait speed (SMD = 1.28, 95%CI 0.36 to 2.19, *p* = 0.006, *I*^*2*^ = 89%), and the timed up and go test (SMD = -0.93, 95% CI − 1.30 to − 0.56, p < 0.0001, *I*^*2*^ = 23%). Resistance training had no effects on appendicular skeletal muscle mass (SMD = 0.25, 95% CI − 0.27 to 0.78, *p* = 0.35, *I*^*2*^ = 68%), skeletal muscle mass (SMD = 0.27, 95% CI − 0.02 to 0.56, *p* = 0.07, *I*^*2*^ = 0%) and leg lean mass (SMD = 0.12, 95% CI − 0.25 to 0.50, *p* = 0.52, *I*^*2*^ = 0%). Old people with sarcopenia of different ages, genders or diagnostic criteria and weights have different gains in muscle mass, handgrip strength, knee extension strength and muscle performance after different intervention duration, frequencies, mode and intensity resistance training.

**Conclusion:**

Resistance training is an effective treatment to improve body fat mass, muscle strength, and muscle performance in healthy older people with sarcopenia.

**Supplementary Information:**

The online version contains supplementary material available at 10.1186/s11556-021-00277-7.

## Introduction

Sarcopenia is an age-related syndrome characterized by a progressive, generalized loss of skeletal muscle mass, combined with a decline in muscle strength and performance [1]. The European Working Group on Sarcopenia in Older People (EWGSOP) reported that the prevalence of sarcopenia in persons aged ≥50 years, ranged from 1 to 29% in community-dwelling populations, 14 to 33% in long-term care settings, and 10% in an acute care setting [2]. In Urumqi (China), China the prevalence of sarcopenia in persons aged ≥60 years ranged from 4.6 to 24.5% depending on the criteria used to define sarcopenia from three organizations (EWGSOP, the International Working Group on Sarcopenia (IGWS), and the Asian Working Group for Sarcopenia (AGWS)) [3]. With the expansion of older populations, sarcopenia-associated morbidity, disability and mortality have made sarcopenia a major global public health problem. Sarcopenia increases the risks of adverse outcomes such as falls and fractures [4] and is associated with cognitive impairment [5], respiratory [6] and sleep disorders [7], poor quality of life, and premature death [8, 9]. This brings a heavy economic burden to societies and families if sarcopenia is untreated [10]. As sarcopenia is a strong indicator for predicting the risk of disability, morbidity, and mortality in middle- and older age people, its treatment and prevention should receive high attention from society and clinical staff [11].

Without effective pharmacological interventions for sarcopenia, non-pharmacological interventions are an effective alternative to decelerate further progression of sarcopenia [12]. Among possible interventions, physical training has been demonstrated as one of the promising method to reduce age-related loss of muscle mass and strength [13]. Of the training modes, resistance training is the most effective in increasing muscle mass and strength in older persons [14]. It promotes improvements in body composition and muscle strength, thereby attenuating the harmful effects of aging [15]. Studies have confirmed the effectiveness of resistance training in older adults with sarcopenia. For example, Jeon et al. [16] showed that a 6-week squat exercise routine could improve hand grip strength (HGS) and knee extensor strength (KES) in older women with sarcopenia. Negaresh et al. [17] demonstrated that an 8-week progressive resistance training program could significantly improve the appendicular skeletal muscle mass index (ASMI) in healthy older men with sarcopenia.

To date, only two meta-analysis studies (Vlietstra et al. [18] and Beckwee et al. [19]) have shown the effectiveness of exercise on muscle mass, muscle strength and muscle performance in older persons with sarcopenia. They noted the results were consistent with other studies showing the benefits of exercise on sarcopenia. However, several factors limit the strengths of the findings. First, an inconsistency of diagnostic criteria and indicators for measuring sarcopenia makes it difficult to study sarcopenia studied in systematic reviews [18, 19]. For example, the sarcopenia diagnostic criteria developed by AGWS [20], EWGSOP-2019 [20], EWGSOP-2010 [21], the Foundation for the National Institutes of Health (FNIH) Sarcopenia Project [22], and others [23–26] differ in the cut-off points of indicator variables (e.g., gait speed (GS), HGS and ASMI) used to define sarcopenia. In addition, the diagnostic criteria may have different combinations of indicator variables in defining sarcopenia (see Table 1). This makes it difficult to evaluate changes in sarcopenia indicator variables consistently in research studies and can reduce the statistical power of meta-analyses studies.

**Table 1:** Different indicators and cut-off points in defining sarcopenia

Diagnosis Criteria	Target district	Cut-off points		
		Muscle mass	Muscle strength	Muscle performance
AGWS [20]	countries from Asia	ASM/height^2^ by DXA: (M: < 7.0 kg/m^2^, F: < 5.4 kg/m^2^); Or ASM/height^2^ by BIA: (M: < 7.0 kg/m^2^, F: < 5.7 kg/m^2^)	HGS: (M:< 28 kg, F:< 18 kg)	GS: < 1.0 m/s; Or 5-STS ≥ 12 s; Or SPPB: ≤9
EWGSOP-2019 [20]	countries from Europe	ASM/height^2^ by DAX or BIA: (M: < 7.0 kg/m^2^, F: < 6 kg/m^2^)	HGS: (M: < 27 kg, F: < 16 kg)	GS: < 0.8 m/s; or 5-STS > 15 s; or SPPB: ≤8; or TUG ≥20s
EWGSOP-2010 [21]	countries from Europe	ASM/height [2] by DXA: (M: < 7.23 kg/m^2^, F: < 5.67 kg/m^2^); or ASM/height^2^ by BIA: (M: < 8.87 kg/m^2^, F: < 6.42 kg/m^2^)	HGS: (M: < 30 kg, F: < 20 kg)	GS: < 1.0 m/s; or SPPB: ≤8
FNIH [22]	/	ASM/BMI by DXA: (M: < 0.789, F: < 0.512)	HGS: (M: < 26 kg, F: < 16 kg)	/
CDC [ 23]	New Mexico	[0.2487(weight) + 0.0483(height)- 0.1584(hip circumference) + 0.0732HGS + 2.5843(sex) + 5.8828] < 2 standard deviations of a young reference population	/	/
Janssen [24]	United States	[(height^2^/BIA-resistance*0.401) + 3.825(gender) + 0.071(age) + 5.102]/body mass*100] < 1 standard deviations of a young reference population	/	/
Tyrovolas [25]	countries from Asia, Africa, Europe, and Latin America	ASM/BMI by BIA, M: ≤0.93 kg/m^2^, F: ≤0.57 kg/m^2^;	HGS: (M: < 30 kg, F: < 20 kg)	GS: (M: 0.95–0.66 m/s; F:0.08–0.48 m/s)
Chung [26]	Korea	ASM/weight^2^*100% by DXA, M: ≤32.5%, F: ≤25.7%	/	/
Fried	United States	weight loss, of ≥10 pounds or, of ≥5% of body weight in prior year	HGS: lowest 20%	GS: slowest 20%
Chen	China	ASM/height^2^ by DXA: (M: < 6.66 kg/m^2^, F: < 5.24 kg/m^2^); or SMM/weight: (M: < 37.15%, F: < 32.26%); or SMM/height^2^: (M: < 8.43 kg/m^2^, F: < 6.80 kg/m^2^)	/	/

ASM: appendicular skeletal muscle mass index; SMM: skeletal muscle mass; BMI: body mass index; M: male; F: female; DXA: dual energy X-ray absorptiometry; BIA: Bioimpedance analysis; HGS: handgrip strength; GS: gait speed; 5-STS: 5 chair sit to stand test; SPPB: the short physical performance battery

Second, the specificity of exercises performed and characteristics of the subjects enrolled in research studies can influence the study outcomes. For example, Jeon et al. [16] found that resistance training could significantly improve appendicular skeletal muscle mass (ASM) in older people without sarcopenia, but the training had no significant effects on ASM in older people with sarcopenia. Thus, sarcopenia may affect the sensitivity and responsiveness of muscles to resistance training. Also, the quality of studies and/or types of exercises performed in research studies can limit the ability to identify changes in sarcopenia indicators in meta-analysis studies. Beckwee et al. [19] showed that resistance training could effectively improve muscle mass, muscle strength, and muscle performance to prevent and treat sarcopenia. However, as an umbrella-review, their study failed to evaluate the quality of the individual randomized controlled trials included in the meta-analysis nor did they analyze the clinical trials to the level of raw data. Vlietstra et al. [18] analyzed the positive effects of different exercise interventions on sarcopenia indicators of KES, HGS, GS, and body fat percentage in healthy older persons with sarcopenia. However, they did not include RCTs using resistance training solely as a treatment mode rendering some of the results as highly heterogeneous. (*I*^*2*^>50%).

No meta-analysis studies have been reported with resistance training as the primary mode of exercise in healthy older people diagnosed with sarcopenia. Thus, it is necessary to integrate more individual randomized controlled trials in a meta-analysis to analyze the effects of resistance training on sarcopenia. In this meta-analysis, we aimed to analyze the results of resistance training on body composition, muscle strength, and muscle performance in healthy older people with sarcopenia to understand the effects of resistance training in treating sarcopenia.

## Material and methods

### Search strategy

This systematic review and meta-analysis was registered (PROSPERO registration number: CRD42020221250), and it was reported in accordance with the Preferred Reporting Items for Systematic Review and Meta-Analysis (PRISMA) statement [27]. We searched the following five electronic databases from January 2010 to October 2020: Pubmed, Embase, Cochrane Library, the China National Knowledge Infrastructure (CNKI), and Wanfang Data. The studies published in English and Chinese were all considered. The following Medical Subject Headings (MeSH) terms and their synonyms were using either singularly or in combination: ‘sarcopenia’, ‘muscle atrophy’, ‘muscle weakness’, ‘muscle loss’, ‘sarcopenic’, ‘resistance training’, ‘resistance exercise’, ‘strength training’, ‘strength training’, ‘weight training’, ‘weight-bearing exercise’, ‘weightlifting’, ‘strength training’, ‘strengthening’,‘resistive exercise’, ‘resistive training’, ‘aged’, ‘frail elderly’, ‘older’, ‘aging’, ‘old’, ‘aged, 80 and over’, and ‘older adults’. The complete search strategy is presented in the supplementary 1.

### Inclusion criteria

Inclusion criteria were as follows: (a) all subjects were diagnosed with sarcopenia according to any established definitions (by a working group on sarcopenia, a certain research or clinical experience); (b) aged>60 years; (c) without other chronic diseases, such as cancer, COPD, diabetes, metabolic syndrome, stroke, and osteoporosis; (d) studies include at least one type of resistance training; (e) a comparison or control group with a no-exercise intervention or that performed other interventions (e.g., education training); (f) outcomes to include body composition (skeletal muscle mass [SMM], leg lean muscle mass [LMM], appendicular skeletal muscle index (ASMI), body fat mass [BFM]), muscle strength (KES, HGS), and muscle performance (GS), and timed up and go [TUG]).

### Exclusion criteria

Exclusion criteria were as follows: (a) articles did not include a full-text description of the study; (b) not in English or Chinese languages; (c) not a randomized, controlled trial; (d) the intervention group received resistance training combined with aerobic training, balance training or nutritional supplementation; and (e) the study presented no extractable data.

### Data extraction

Two reviewers (NC and XH) independently screened the title and abstract of the studies to exclude those that failed to meet the inclusion criteria and/or that met the exclusion criteria. The remaining full-text studies were evaluated according to inclusion and exclusion criteria. If there was a disagreement between the two reviewers, a third reviewer (YL) participated in discussing the issue until the disagreement was resolved. Two reviewers (NC and XH) independently extracted the characteristics of subjects (e.g., demographic characteristics), resistance training intervention (e.g., modality, intensity, frequency, and duration), and the outcome using a standard extraction form developed for this study. If a study was a multiple-arm intervention, we extracted only the data of intervention groups receiving resistance training. We also contacted the authors of the included studies for raw data that were not shown in the original papers.

### Quality assessment

Two reviewers (NC and XH) independently assessed the methodological quality of the studies using the Physiotherapy Evidence Database (PEDro) scale [28]. The scale assesses the following 11 characteristics: eligibility criteria; random allocation; concealment allocation; baseline similarity; blinding of the subjects, therapists, and assessors; measures of at least one key outcome from more than 85% of subjects; ‘intention to treat’ analysis; between-group statistical comparisons; and point measures or measures of variability. Each characteristic was rated 0 (characteristic was not met the criteria) to 1 (characteristic met the criteria) for each study. The higher the total score, the higher the quality of the study. If there was a disagreement between the two reviewers, the third reviewer (YL) participated in the evaluation and discussion.

### Statistical analysis

All data were analyzed using the Review Manager (RevMan 5.4; Cochrane, Lindon, UK). We used *I*^*2*^ statistic to evaluate heterogeneity among the included studies for each outcome. To calculate pooled effect sizes, inverse variances were used as statistical method, fixed-effect models (*I*^*2*^ < 50%) and random-effect models (*I*^*2*^ *>* 50%) were conducted as analysis model and 95% confidence intervals (CI) were calculated as the effect measure reported as standardized mean differences (SMD). To explore the influence of moderator variables on muscle mass, muscle strength and muscle performance, we performed subgroup analyses to assess the potential effects of different moderator. Due to the limited number of articles included, we integrated the outcome of SMM, ASMI, and LLM into muscle mass, TUG and GS into muscle performance, and used HGS and KES as separate outcomes for subgroup analysis. The moderator variables of age; gender; sarcopenia diagnostics criteria; obesity; intervention duration; frequency; mode; intensity were included in the subgroup analysis. All data were continuous variables and *P* < 0.05 was considered to a statistical significance. We contacted the authors of included studies if we could not extract valid mean values or standard deviations from the paper. If the authors contacted did not reply, we excluded their studies or related indicators.

## Results

### Study selection

**Fig. 1:**
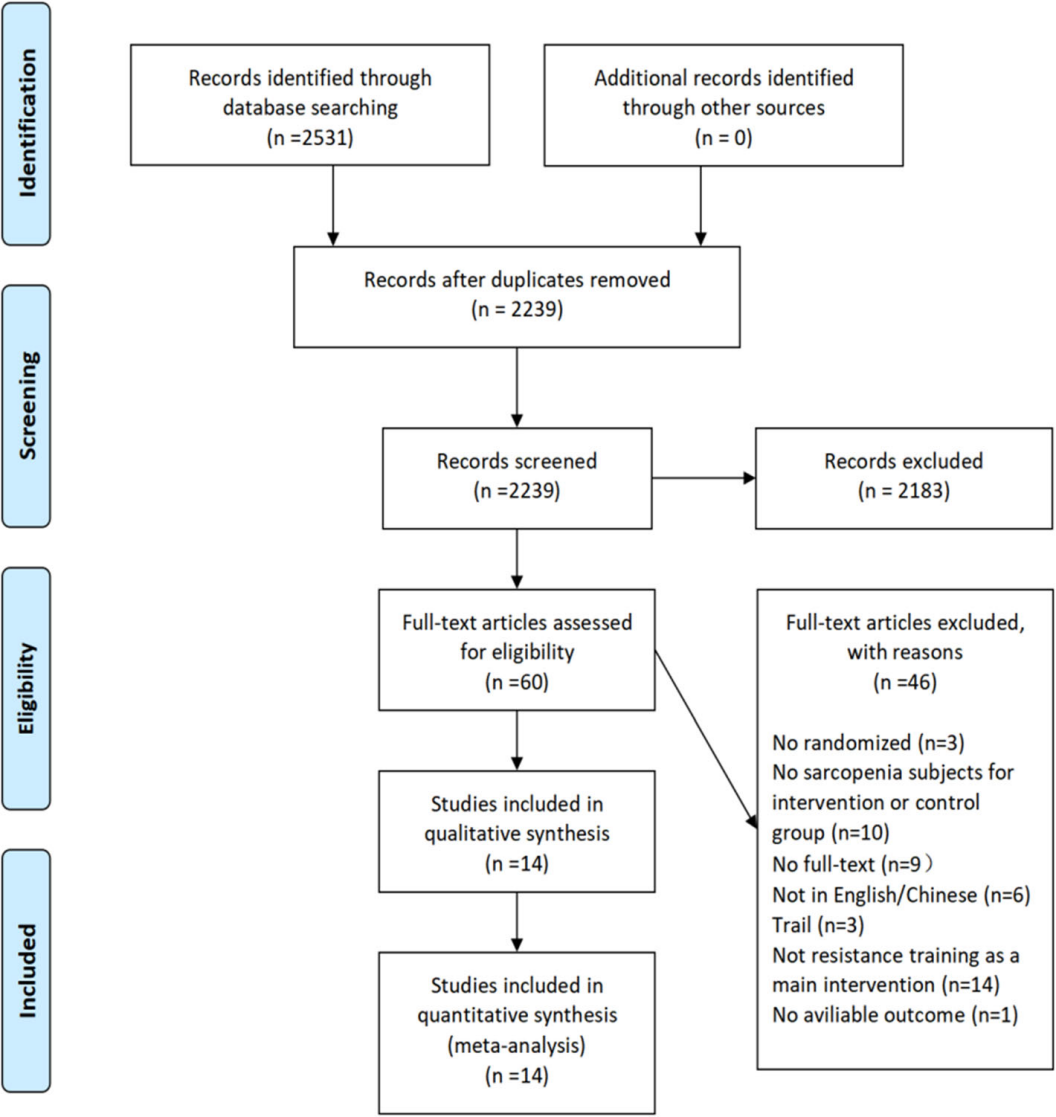
Flow of screening and selecting process according to Preferred Reporting Items for Systematic Reviews and meta-analysis (PRIAMA)

Our search resulted in 2531 records in databases using keywords according to the search strategy. After removing the duplicate records, 2239 records remained. Examination of the titles and abstracts resulted in excluding 2183 articles that did not meet the inclusion and exclusion criteria. Of the remaining 60 articles, we reviewed the full texts and further excluded 49 articles that did not meet the inclusion and exclusion criteria. Finally, we included 14 studies that met the inclusion and exclusion criteria in the systematic review meta-analysis (see Fig. 1).

### Study characteristics

The characteristics of the 14 studies included in the meta-analysis is shown in Table 2. The meta-analysis included 561 older people with sarcopenia, 292 (52%) of whom received various modes of resistance training. Seven studies included both genders, six included only females, and one had no sex listed. The diagnostic criteria for sarcopenia in the 14 studies was adopted from the following: EWGSOP [20, 21] (4 studies), AWGS [20] (3 studies), and Centers for Disease Control and Prevention [23] [CDC] (1 study). The remaining four studies used diagnostic criteria developed for their studies [24–26, 29, 30]. The resistance training in seven studies were performed with the following exercise modes: kettlebells (1 study), dumbbells (1 study), suspension bands (1 study), elastic bands (4 studies), weight loads (3 study), weight machines (3 studies) and body weight (3 studies). Three studies used more than one mode of resistance training. The training movements in 11 of the studies focused on the muscle groups of the upper and lower limbs, and 3 study focused only on the lower limbs. The training intensity ranged from 40 to 80% of 1-repetition maximum (1RM), of which 7 studies adopted progressive resistance training methods. The remaining studies used other resistance training methods. Training frequency varied from 1 to 3 times per week and the program duration ranged from 8 to 36 weeks. For the interventions in the control groups, ten studies had subjects maintain their usual lifestyle without any exercise intervention, three studies provided patient education and one study provided a postural intervention.

**Table 2:** Characteristics of Included Studies

Study	Sample size (RTG/CG)	Gender (n:male /female)	Age^**#**^ (RTG/CG)	Sarcopenia diagnostics (Indicator, Cut-points, Source)	Intervention				Control group	Outcome
					Mode	Training movement	Intensity	Duration times/week;(total weeks)		
Chen et al. [31] (2018)	17/16	0/33	66.7 ± 5.3 /68.3 ± 2.8	ASM/height^2^ by BIA < 5.7 kg/m^2^; HGS, < 18 kg, GS < 0.8 m/s (AWGS)	Kettleball	Swing, deadlift, goblet squat, squat lunge, row, single arm row, biceps curl, triceps extension, two-arm military press, Turkish get up, and comprehensive dynamic workout	3sets/8-12reps 60–70%1RM	2(8)	Non-exercise	ASMI SMM BFM HGS
Cebria Iranzo et al. [32] (2018)	11/17	18/29	82.8 ± 9.1 /81.2 ± 5.4	ASM/BMI by BIA, M: ≤0.93 kg/m^2^, F:≤0.57 kg/m^2^; GS, M: 0.95–0.66 m/s; F: 0.08–0.48 m/s (Tyrovolas)	Dumbbell and ankle/wrist weights	Ankle flexion/extension, knee extension, hip flexion/ abduction/adduction, handgrip, wrist flexion/extension, forearm pronation/supination, elbow/ flexion/extension, and shoulder flexion/extension /adduction/abduction	40–60% 1RM	3(12)	Non-exercise	ASMI HGS KES GS
Wei-Hua et al. [33] (2020)	41/30	63/73	70.04 ± 9.86 /68.54 ± 10.62	ASM/height^2^ by BIA, M: < 7 kg/m^2^, F: < 5.7 kg/m^2^; HGS, M: < 26 kg; F: < 18 kg; GS < 0.8 m/s (AWGS)	Weight machine	Knee extension/flexion, chest press, shoulder press, and low back muscle training	30 min	2(24)	Education	ASMI HGS GS
Piastra et al. [34] (2018)	35/37	Not listed	69.9 ± 2.7 /70.0 ± 2.8	SMM/height^2^ by BIA, M: ≤10.75 kg/m^2^; F: ≤6.75 kg/m^2^; HGS, M: < 30 kg, F: < 20 kg (EWGSOP-2019)	Weight loads	30 min muscle toning for different muscular districts (primarily abdominal and both lower and upper limbs)	Low/moderate	2(36)	Postural training	SMM HGS
Vikberg et al. [35] (2019)	36/34	44/47	70.9 ± 0.28 /70.0 ± 0.29	appendicular lean muscle mass/height^2^ by DXA, M: ≤7.29 kg/m^2^; F: ≤5.53 kg/m^2^ (EWGSOP-2010)	Suspension band and body weight	Squats, calf raises, chair stands, half lunges, biceps rowing, push-ups, and bridge	Week 1: 2set/10reps; Week 2–4: 3set/10reps; Week 5–7: 4set/10reps	1(10)	Non -exercise	BFM LLM HGS TUG
Bellomo et al. [36] (2013)	10/10	20/0	Average: 70.9 ± 5.2	muscle mass/height^2^ by DXA,< 2 std. dev of a young reference population (CDC)	Weight machine	Leg press and extension	Week 1–4: 1set/15reps; Week 5–8: 3set/12reps; Week 9–12: 3sets/6-8reps	2(12)	Education	KES
Zhao et al. [37] (2016)	6/6	12/0	65.8 ± 2.48/66 ± 3.12	ASM/height^2^ by BIA, M: < 7 kg/m^2^; HGS < 26 kg, GS < 0.8 m/s (AWGS)	Elastic band and body weight	Squat on the bench for lower limbs exercise, resist external loads for upper limbs exercise	60–80%1RM 3 sets 40 min	3(8)	Non-exercise	ASMI HGS
Huang et al. [38] (2017)	18/17	0/35	68.89 ± 4.91/69.53 ± 5.09	SMM/weight^2^*100% by BIA < 27.6% (Janssen)	Elastic band	Exercises for training major muscle groups (shoulders, arms, lower limbs, chest, and abdomen)	3 sets/10 reps	3(12)	40-min lesson about exercise	BFM
Chen et al. [39] (2017)	15/15	5/25	68.9 ± 4.4/68.6 ± 3.1	ASM/weight^2^*100% by BIA, M: ≤32.5%, F: ≤25.7% (Chung)	Weight machine	Shoulder presses, bicep curls, triceps curls, bench presses, deadlifts, leg swings, squats, standing rows, unilateral rows, and split front squats	60–70% 1RM 3 sets/8–12 reps	2(8)	Non-exercise	SMM BFM HGS KES
Liao et al. [40] (2018)	33/23	0/55	66.67 ± 4.54/68.32 ± 6.05	ASM/weight^2^*100% by BIA, M: ≤32.5%, F: ≤25.7% (Chung)	Elastic band	Seated chest press, seated row, seated shoulder press, knee extension, knee flexion, hip flexion, and hip extension	3 sets/10 reps	3(12)	Non-exercise	SMM HGS KES GS TUG
Liao et al. [41] (2017)	25/21	0/46	66.39 ± 4.49/68.42 ± 5.86	SMM/height^2^ by DXA, F: ≤7.15 kg/m^2^ (EWGSOP-2010)	Elastic band	Seated chest press, seated row, seated shoulder press, Concentric–eccentric hip circumduction, leg press, and leg curl	3sets /10 reps	3(12)	Non-exercise	LLM BFM HGS KES GS TUG
Hamaguchi et al. [42] (2017)	7/8	0/15	60.4 ± 2.7/60.6 ± 2.3	SMM/height^2^ by DXA < 6.12 kg/m^2^ (EWGSOP-2010)	Weight loads	Front lunge, side lunge, calf raise, and toe raise	8 sets /3 reps	2(6)	Non-exercise	KES HGS
Vasconcelos et al. [43] (2016)	14/14	0/28	72 ± 4.6/72 ± 3.6	HGS < 21 kg (Fried)	Elastic band	Knee exercises, hip exercises, and mini-squats	2-3sets/12 reps (40–60%1RM) for knee exercises 2-3sets/12reps (1-3 kg) for hip exercises 2-3sets/10reps (1-3 kg) for mini-squats	2(10)	Non-exercise	KES GS
Chiu et al. [29] (2018)	24/21	35/35	79.64 ± 7.36/80.15 ± 8.26	SMM/weight*100% by BIA,M: ≤37.15%, F: ≤32.26%% (Chen)	Sandbag or grip ball	Upper body exercises included training that targeted the biceps, deltoids, grip, and pinch. Lower extremities consisted of leg extension, leg flexion, calf raises, stepping forward and sideward,	3sets/4-10reps	2(12)	Non-exercise	ASMI HGS

1RM: one repetition maximum; reps: repetitions; M: male; F: female; LLM: leg lean mass; ASM: appendicular skeletal muscle mass; ASMI: appendicular skeletal muscle mass index; SMM: skeletal muscle mass; SMI:skeletal muscle mass; BFM: body fat mass; HGS: hand grip strength; KES: knee extension strength; GS: gait speed; DXA: dual energy X-ray absorptiometry; BIA: Bioimpedance analysis; AWGS: Asian Working Group for Sarcopenia; CDC: Centers for Disease Control and Prevention; EWGSOP: European Working Group on Sarcopenia in Older People; *RTG: resistance training group; CG:control group; #Age is presented as Mean ± Standard Deviation

### Quality assessment

The domain scores of each study for the quality assessment are shown in Table 3. Out of a maximum of 10 points, two studies scored 5 points, five studies scored 6 points, 1 study scored 7 points, and 3 studies scored 8 points. All studies reported random allocation, baseline similarity, and point measures. Five studies reported concealment allocation and 10 studies reported measures of at least one key outcome in more than 85% of the subjects. Six studies performed intention-to-intention analysis and 10 studies performed group comparisons. Six studies mentioned assessor blinding and one study mentioned therapists blinding. There was no study that blinded the subjects.

**Table 3:** PEDro Criteria and Scores of Included Studies

study	Random allocation	Concealed allocation	Baseline similar	Blinding (subject)	Blinding (therapists)	Blinding (Assessor)	Measure for>85%	Intention-to-Treat Analysis	Group Comparison	Point measures	Total score (0–10)
Chen et al. (2018)	1	0	1	0	0	0	1	1	1	1	6
Cebria I Iranzo et al. (2018)	1	1	1	0	1	1	0	0	1	1	7
Piastra et al. (2018)	1	0	1	0	0	0	1	0	1	1	5
Wei-Hua et al. (2020)	1	0	1	0	0	0	1	1	1	1	6
Vikberg et al. (2019)	1	1	1	0	0	0	1	0	1	1	6
Bellomo et al. (2013)	1	0	1	0	0	1	1	0	0	1	5
Zhao et al. (2016)	1	0	1	0	0	0	1	1	1	1	6
Huang et al. (2017)	1	1	1	0	0	1	1	1	1	1	8
Chen et al. (2017)	1	0	1	0	0	1	1	0	1	1	6
Liao et al. (2018)	1	1	1	0	0	1	1	1	1	1	8
Liao et al. (2017)	1	1	1	0	0	1	1	1	1	1	8
Hamaguchi et al. (2017)	1	0	1	0	0	0	1	0	1	1	5
Vasconcelos et al. (2016)	1	1	1	0	0	1	1	1	1	1	8
Chiu et al. (2018)	0	0	0	0	0	0	1	0	1	1	3

PEDro: physiotherapy Evidence Database; 1: met the criteria; 0: not met the criteria

### Outcomes

#### Body composition

**Fig. 2:**
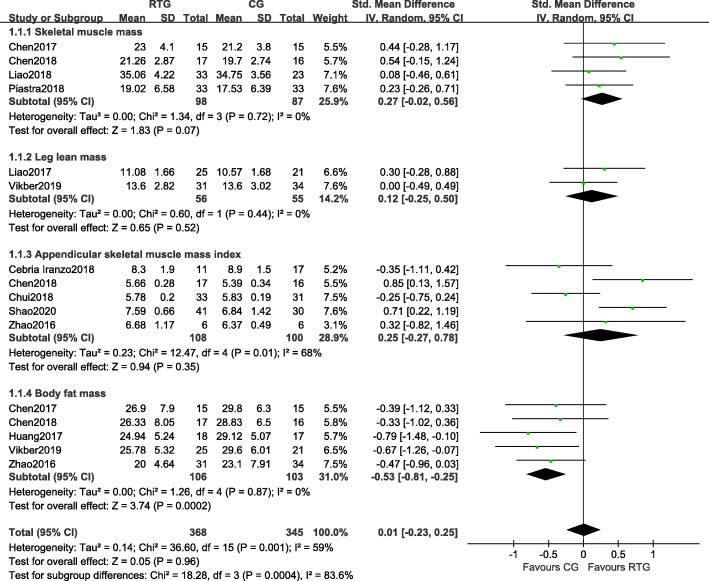
Forest plots of the comparison of the resistance training group (RTG) versus the control group (CG) on a: skeletal muscle mass (SMM); b: leg lean mass (LLM); c: appendicular skeletal muscle mass index (ASMI); CI: confidence interval; SD: standard deviation

Eleven of fourteen studies assessed the effects of resistance training on body composition. There were two main outcomes: muscle mass (SMM, LLM, ASMI) and BFM (Fig. 2).

Of these studies, four measured the effects of resistance training on SMM. There were no significant differences in SMM between the resistance training group and the control group (SMD = 0.27, 95% CI − 0.02 to 0.56, *p* = 0.07, *I*^*2*^ = 0%). Two studies measured the effects of resistance training on LLM. No significant differences were observed in LLM between the resistance training and control groups (SMD = 0.12, 95% CI − 0.25 to 0.50, *p* = 0.52, *I*^*2*^ = 0%). Five studies measured the effects of resistance training on ASMI. Compared with the control group, there was no significant increase in ASMI in the resistance training group (SMD = 0.25, 95% CI − 0.27 to 0.78, *p* = 0.35, *I*^*2*^ = 68%). Five studies measured the effects of resistance training on BFM. Compared with the control group, there was a significant decrease in BFM in the resistance training group (SMD = -0.53, 95% CI − 0.81 to − 0.25, *p* = 0.0002, *I*^*2*^ = 0%).

#### Muscle strength

**Fig. 3:**
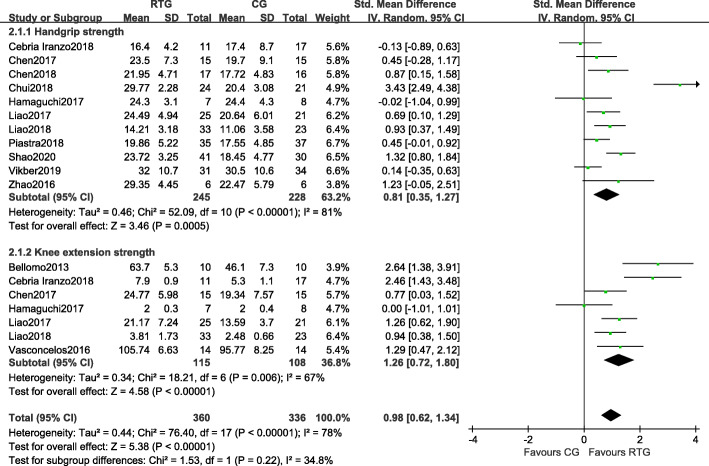
Forest plots of the comparison of the resistance training group (RTG) versus the control group (CG) on a: hand grip strength (HGS) and b: knee extension strength (KES). CI: confidence interval; SD: standard deviation

Thirteen studies measured the effects of resistance training on muscle strength for HG and KES (Fig. 3). Of these studies, eleven measured the effects of resistance training on HGS. Compared with the control group, there was a significant increase in HGS in the resistance training group (SMD = 0.81, 95%CI 0.35 to 1.27, *p* = 0.0005, *I*^*2*^ = 81%). Seven studies measured the effects of resistance training on KES. Compared with the control group, there was a significant increase in KES in the resistance training group (SMD = 1.26, 95% CI 0.72 to 1.80, *p* < 0.0001, *I*^*2*^ = 67%).

#### Muscle performance

**Fig. 4:**
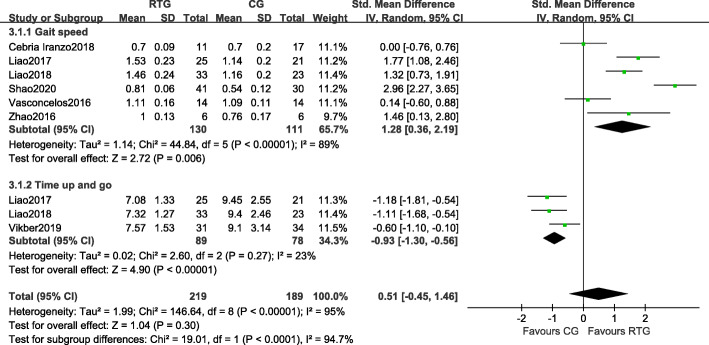
Forest plots of the comparison of the resistance training group (RTG) versus the control group (CG) on a: gait speed (GS); b: time up and go (TUG). CI: confidence interval; SD: standard deviation

Six studies measured the effects of resistance training on muscle performance for GS and the TUG (Fig. 4). Of these studies, six assessed the effects of resistance training on GS. Compared with the control group, there was a significant increase in GS in the resistance training group (SMD = 1.28, 95%CI 0.36 to 2.19, *p* = 0.006, *I*^*2*^ = 89%). Three studies measured the effects of resistance training on the TUG. Compared with the control group, there was a significant decrease in time in the resistance training group (SMD = -0.93, 95% CI − 1.30 to − 0.56, *p* < 0.0001, *I*^*2*^ = 23%).

#### Moderator variables

**Fig. 5:**
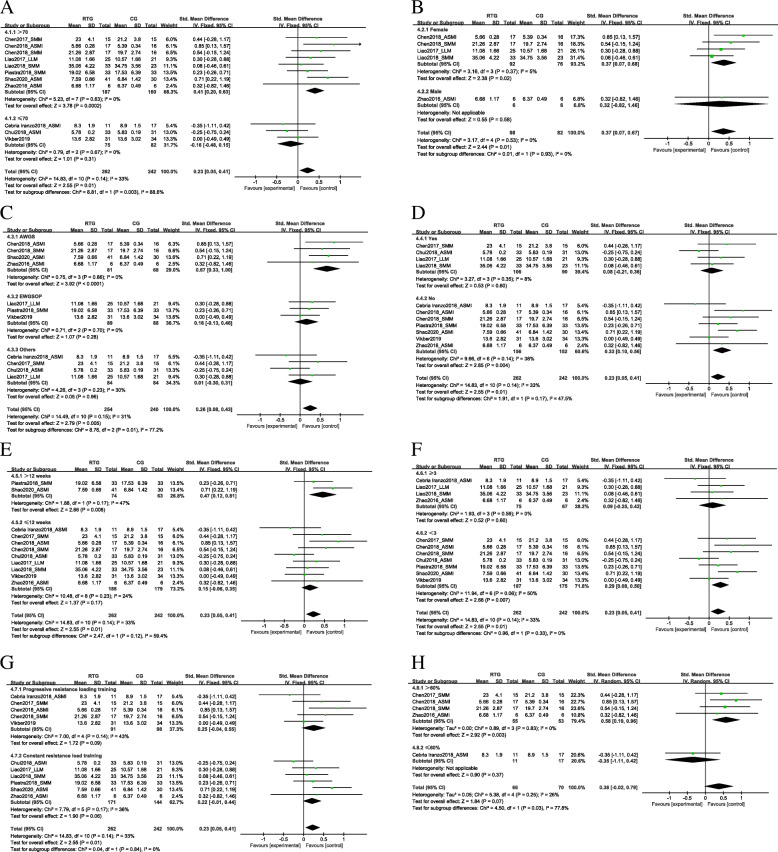
Forest plots of RCTs investigating of the effect of the resistance training group (RTG) versus the control group (CG) on muscle mass according to a: Age; b: Gender; c: Sarcopenia diagnostics criteria; d: Obesity; e: Intervention duration; f: Frequency; g: Mode; h: Intensity; CI: confidence interval; SD: standard deviation

Muscle mass: Subgroup analysis (Fig. 5) showed the effect of resistance training on muscle mass according to the participants features, resistance training protocol. Muscle mass significant increase in aged > 70 (SMD = 0.41, 95% CI 0.2 to 0.63, *p* = 0.0002), female (SMD = 0.37, 95% CI 0.07 to 0.68, *p* = 0.02), with AWGS sarcopenia diagnostics criteria (SMD = 0.67, 95% CI 0.33 to 1.00, *p* < 0.0001), normal weight (SMD = 0.33, 95% CI 0.10 to 0.56, *p* = 0.004) subjects. Concerning resistance training protocol, a greater effect on muscle mass was observed when resistance training included < 3 times per week (SMD = 0.29, 95% CI 0.08 to 0.50, *p* = 0.007), with a total duration ≥12 weeks (SMD = 0.47, 95% CI 0.12 to 0.81, *p* = 0.008), and using > 60% 1RM intensity (SMD = 0.58, 95% CI 0.19 to 0.96, p = 0.003).

Muscle strength: In Table 4, HGS significant increase in aged ≤70 (SMD = 0.84, 95% CI 0.53 to 1.15, *p* < 0.0001), female (SMD = 1.2, 95% CI 0.88 to 1.52, *p* = 0.005), with AWGS sarcopenia diagnostics criteria (SMD = 1.17, 95% CI 0.77 to 1.57, *p* < 0.0001), have obesity (SMD = 1.32, 95% CI 0.27 to 2.38, *p* = 0.01) subjects. Concerning resistance training protocol, a significant increased in HGS was both observed when resistance training included < 3 times per week (SMD = 0.62, 95% CI 0.39 to 0.85, *p* = 0.04) or ≥ 3 times per week (SMD = 0.65, 95% CI 0.3 to 1, *p* = 0.02), and with a total duration > 12 weeks (SMD = 0.88, 95% CI 0.03 to 1.72, p = 0.04) or ≤ 12 weeks (SMD = 0.74, 95% CI 0.07 to 1.4, *p* = 0.03). A greater effect on HGS was observed performed as a constant resistance loading training (SMD = 0.97, 95% CI 0.72 to 0.82, *p* = 0.0007) and using > 60% 1RM intensity (SMD = 4.66, 95% CI 2.1 to 7.22, *p* < 0.0001).

**Table 4:** Influence of moderator variables in the effect of resistance training on Handgrip strength, Knee extension strength, and Muscle performance

Variable	Subgroup	Studies	n	Effect Size with 95% Confidence Interval	Heterogeneity				Test overall effects. Z(p)	Test for Subgroup Difference. Chi^2^ (p)
					Tau^2^	Chi^2^	p	*I*^*2*^		
Handgrip strength (HGS)										
Age (years)	> 70	3	138	0.91 [−1.21, 3.03]	3.36	52.59	< 0.0001	96	0.84 (0.4)	0.01 (0.94)
	≤70	8	335	0.84 [0.53, 1.15]	0.08	11.88	0.1	41	5.28 (< 0.0001)	
Gender	Female	5	195	1.2 [0.88, 1.52]	0.87	28.71	< 0.0001	86	2.82 (0.005)	0.00 (0.95)
	Male	1	12	1.23 [−0.05, 2.51]	Not applicable				1.88 (0.06)	
Sarcopenia diagnostics criteria	AWGS	3	116	1.17 [0.77, 1.57]	0.00	1	0.61	0	5.71 (< 0.0001)	3.86 (0.14)
	EWGSOP	4	198	0.27 [−0.02, 0.55]	0.49	18.72	0.0003	84	0.81 (0.42)	
	Other	4	159	0.93[0.58, 1.29]	1.52	36.12	< 0.0001	92	1.76 (0.08)	
Obesity	Yes	4	177	1.32 [0.27, 2.38]	1.03	28.56	< 0.0001	89	2.45 (0.01)	2.11(0.15)
	No	7	296	0.41[0.17,0.65]	0.43	29.56	< 0.0001	80	1.52 (0.14)	
Intervention duration (weeks)	> 12	2	143	0.88[0.03, 1.72]	0.31	5.82	< 0.0001	83	2.04(0.04)	0.06(0.8)
	≤12	9	330	0.74[0.07, 1.4]	0.86	60.18	< 0.0001	87	2.18 (0.03)	
Frequency (days/week)	≥3	4	142	0.65[0.3, 1]	0.13	5.83	0.12	49	2.42 (0.02)	0.17 (0.68)
	<3	7	331	0.62[0.39,0.85]	0.98	62.12	< 0.0001	90	2.09 (0.04)	
Mode	Progressive resistance load training	4	156	0.04[−0.28, 0.36]	0.29	10.54	0.01	72	0.47 (0.64)	4.47 (0.03)
	Constant resistance load training	7	317	0.97[0.72,0.82]	0.59	37.1	< 0.0001	84	7.8 (0.0007)	
Intensity(1RM)	>60%	3	75	4.66[2.1, 7.22]	0.00	0.7	0.7	0	3.56 (0.0004)	4.12 (0.04)
	≤60%	1	28	-1[−5.82, 3.82]	3.61	Not applicable			0.41 (0.68)	
Knee extension strength (KES)										
Age (years)	>70	3	76	2.05[1.16, 2.94]	0.34	4.5	0.11	56	3.92(< 0.0001)	5.58(0.02)
	≤70	4	147	0.86[0.43, 1.28]	0.06	4.38	0.22	32	4.51(< 0.0001)	
Gender	Female	4	145	0.96[0.49, 1.43]	0.09	4.91	0.18	39	4.03(< 0.0001)	5.97(0.01)
	Male	1	20	2.64[1.38, 3.91]	Not applicable				4.09(< 0.0001)	
Sarcopenia diagnostics criteria	AWGS	Not applicable								1.3(0.25)
	EWGSOP	2	61	0.7[−0.54, 1.93]	0.61	4.26	0.04	77	1.11(0.27)	
	Other	5	162	1.49[0.82, 2.16]	0.38	12.66	0.01	68	4.38(< 0.0001)	
Obesity	Yes	4	160	1.05[0.72, 1.39]	0.00	1.43	0.7	0	6.15(< 0.0001)	0.49(0.49)
	No	3	63	1.56[0.94, 2.19]	2.00	14.81	0.0006	86	1.91(0.06)	
Intervention duration (weeks)	>12	Not applicable								Not applicable
	≤12	7	223	1.26[0.72, 1.8]	0.34	18.21	0.006	67	4.58(< 0.0001)	
Frequency (days/week)	≥3	3	130	1.44[0.70, 2.18]	0.29	6.45	0.04	69	6.42(0.0001)	0.3(0.58)
	<3	4	93	1.11[0.21, 2.01]	0.6	11.03	0.01	73	2.43(0.02)	
Mode	Progressive resistance load training	4	106	1.70[0.83, 2.57]	0.55	10.33	0.02	71	6.47(0.0001)	2.5(0.11)
	Constant resistance load training	3	117	0.85[0.25,1.45]	0.15	4.27	0.12	53	2.77(0.006)	
Intensity(1RM)	>60%	1	30	5.43[0.55, 10.31]	Not applicable				2.18(0.03)	0.01(0.94)
	≤60%	2	56	5.75[−1.39, 9.63]	23.09	6.67	0.010	85	1.58(0.11)	
Muscle Performance										
Age (years)	>70	3	100	−0.21[−1.00, 0.58]	0.35	7.23	0.03	72	0.53(0.06)	1.12(0.29)
	≤70	5	275	0.75[−0.84, 2.33]	3.17	126.31	< 0.0001	95	0.92(0.36)	
Gender	Female	5	232	0.18[−1.03, 1.39]	1.80	71.40	< 0.0001	94	0.3(0.77)	Not applicable
	Male	Not applicable								
Sarcopenia diagnostics criteria	AWGS	1	71	2.96[2.27, 3.65]	Not applicable				8.44 (< 0.0001)	23.48(91.5)
	EWGSOP	3	138	−0.12[−1.9, 1.66]	2.36	45.63	< 0.0001	96	0.13(0.89)	
	Other	4	166	0.15[−0.97, 1.27]	1.18	33.6	< 0.0001	91	0.26(0.80)	
Obesity	Yes	5	232	0.18[−1.03, 1.39]	1.80	71.4	< 0.0001	94	0.3(0.53)	0.18(0.67)
	No	3	143	0.76[−1.63, 3.15]	4.32	69.87	< 0.0001	97	0.62(0.53)	
Intervention duration (weeks)	>12	1	71	2.96[2.27, 3.65]	Not applicable				8.44(< 0.0001))	25.31(< 0.0001)
	≤12	7	304	0.03[−0.88, 0.94]	1.4	81.18	< 0.0001	93	0.07(0.95)	
Frequency (days/week)	≥3	5	230	0.21[−1.02, 1.43]	1.85	71.5	< 0.0001	94	0.33(0.74)	0.15(0.70)
	<3	3	145	0.72[−1.61, 1.46]	4.12	70.72	< 0.0001	97	0.61(0.54)	
Mode	Progressive resistance load training	3	100	−0.2[−1.00, 0.58]	0.35	7.23	0.03	72	0.53(0.60)	1.12(0.29)
	Constant resistance load training	5	275	0.75[−0.84, 2.33]	3.17	126.31	< 0.0001	97	0.92(0.36)	
Intensity(1RM)	>60%	Not applicable								Not applicable
	≤60%	2	72	−0.37[−1.53, 0.79]	0.57	5.22	0.02	81	0.63(0.53)	

In Table 4, a significant increased in KES was both observed in aged > 70 (SMD = 2.05, 95% CI 1.16 to 2.94, p < 0.0001) or ≤ 70 (SMD = 0.86, 95% CI 0.43 to 1.28, p < 0.0001), female (SMD = 0.96, 95% CI 0.49 to 1.43, p < 0.0001) or male (SMD = 2.64, 95% CI 1.38 to 3.91, p < 0.0001). KES significant increase in with other sarcopenia diagnostics criteria (SMD = 1.49, 95% CI 0.82 to 2.16, p < 0.0001) and have obesity (SMD = 1.05, 95% CI 0.72 to 1.39,p < 0.0001) subjects. Concerning resistance training protocol, a significant increased in KES was both observed when resistance training included < 3 times per week (SMD = 1.11, 95% CI 0.21 to 2.01, *p* = 0.02) or ≥ 3 times per week (SMD = 1.44, 95% CI 0.7 to 2.18, *p* = 0.00001), progressive resistance load training (SMD = 1.70, 95% CI 0.83 to 2.57, p = 0.00001) or constant resistance load training (SMD = 0.85, 95% CI 0.25 to 1.45, *p* = 0.0006). A greater effect on KES was observed in a total duration ≤12 weeks (SMD = 1.26, 95% CI 0.72 to 1.8, *p* = 0.008) and using > 60% 1RM intensity (SMD = 5.43, 95% CI 0.55 to 10.31, *p* = 0.03).

Muscle performance: In Table 4, muscle performance only significant increase in aged > 70 (SMD = -0.21, 95% CI − 1 to 0.58, *p* = 0.06), with AWGS sarcopenia diagnostics criteria (SMD = 2.96, 95% CI 2.27 to 3.65, *p* < 0.0001) subjects. Concerning resistance training protocol, a greater effect on muscle mass was only observed when resistance training with a total duration ≥12 weeks (SMD = 2.96, 95% CI 2.27 to 3.65, p < 0.0001).

## Discussion

High quality evidence on the effects of resistance training in healthy older people with sarcopenia is limited. To address this, we combined 14 randomized controlled trials to explore the effects of resistance training on body composition, muscle strength, and muscle performance in older people with sarcopenia. Pooled analyses showed that, compared with no-exercise or non-exercise activities in older people with sarcopenia, resistance training had significant beneficial effects on the body fat mass, handgrip strength, knee extension strength, gait speed, and time up and go. But have no significant effect on skeletal muscle mass, leg lean mass, and appendicular skeletal muscle mass index. These results indicate that resistance training has the potential to favorably influence in outcomes related to the sarcopenia.

According to a recent review, resistance training has been shown to increase muscle protein synthesis, increase the size of type 1 and type 2 muscle fibers, and lead to overall improvements in muscle strength and physical performance in older people with sarcopenia [44]. Fundamentally, muscle mass is related to body size. Therefore, when quantifying muscle mass, the absolute level of SMM or ASM can be adjusted according to body size in different ways [45]. ASMI, defined as appendicular skeletal muscle mass/height^2^ [ASM/m^2^], is frequently used in studies to diagnose and evaluate sarcopenia. This measurable method depends upon on ASM, which is measured by dual energy X-ray absorptiometry (DXA) or bioimpedance analysis (BIA). However, our meta-analysis differs in that we failed to show any effects of resistance training on SMM, LLM and ASMI in older people with sarcopenia. This finding is consistent with Vlietstra’s meta-analysis that showed no effects of exercise interventions on muscle mass in older people with sarcopenia [11]. However, their study only includes four articles related to the effect of resistance training. In a meta-analysis reported by Peterson and Gordon, resistance training significantly increased muscle mass in older people [46]. A meta-analysis conducted by Martins indicated that exercise interventions had no effect on ASMI in older people [47]. We think this inconsistency in findings might be caused by the differences in resistance training parameters. For example, increases in muscle mass in older adults have been observed in studies using a longer exercise intervention period (at least 6 months) as compared with shorter exercise intervention periods [48]. While our study showed resistance training failed to increase muscle mass, there were positive effects on muscle strength and muscle performance. It is demonstrate that neural mechanisms and muscular innervation, such as adaptations in activation, synchronization, and rate coding, rather than muscular hypertrophy, are the most likely reasons of increased muscle strength [49]. For novices, improvements in muscle strength during the first 8 weeks of resistance training programs are usually attributed to improved neural adaptations rather than changes in muscle structural [50]. Consistent with our results, Leandro et al. indicated that the improvement in muscle performance by resistance training was associated with increased muscular strength but not with changes in muscle mass or body fat in older women. They concluded that a short training duration (8 weeks) failed to improve muscle mass and therefore, could not improve muscle performance [51]. Accordingly, we speculate that differences in training modes may have different effects on muscle mass. Therefore, we performed a subgroup analysis to assess the effects of exercise protocols on muscle mass, and we found that resistance training performed 1–2 times per week at an intensity>60% 1RM for an intervention duration ≥12 weeks resulted in greater gains in muscle mass.

In relation to the effects of resistance training on BFM, sarcopenia often is associated with obesity due to changes in endocrine function and a lack of physical activity leading to reduced muscle mass and strength. Older people with sarcopenia tend to show high levels of body fat and visceral fat [52]. When sarcopenia is combined with obesity, it is called sarcopenic obesity (SO). Our study found that resistance training could significantly decrease the BFM in older people with sarcopenia. Consistent with our finding, the meta-analysis by Hsu et al. showed that resistance training could significantly decrease BFM in older people with SO [53].

Muscle strength and performance are important for active living and independence in older people as both strength and performance decrease more rapidly than muscle mass in older people, especially in women [54]. The HGS is a useful test for evaluating overall muscle strength as it has a strong relationship with lower limb strength. The KES test also reflects the muscle strength of lower limbs and is related to locomotion, activities of daily living, and the risk of falling accidents [55]. Results from our meta-analysis showed that resistance training significantly improved HGS and KES scores in older people with sarcopenia. Consistent with our study, a meta-analyses by Peterson et al. showed that resistance training significantly improved KES scores in healthy older people with [11] and without [56] sarcopenia. In contrast, a meta-analysis by Vlietstra et al. showed no changes in the HGS scores following an exercise intervention in healthy older people with sarcopenia [11]. A meta-analysis by Grgic et al., also showed no effects of resistance training on HGS scores in very old people [57]. Our research also proves that only female people younger than 70 years old have gained significant improvement after resistance intervention. A lack of improvement in HGS scores in some studies might be due to adaptations to resistance training that are highly specific and dependent on the mode and dose of exercise [58]. Some of the studies included in our meta-analysis involved resistance movements that specifically improved handgrip strength, neither of which were included in the two previously mentioned meta-analysis studies that failed to show improvements in the HGS scores [39]. It should be noted that, while hand grip strength may reflect overall body strength, increases in hand grip strength following resistance training are minimal in older people [40].

Muscle performance is a multidimensional concept, defined as an objectively measured whole body function related with mobility that involves many organs and systems of the body” [59]. GS and the TUG are the most commonly used tests to evaluate the muscle performance of older people. Perera et al. defined clinical thresholds for increases in GS following a resistance training program in older people as small (≈ 0.05 m/s) and substantial (≈ 0.10 m/s) [60]. Our research showed that resistance training significantly improved GS and TUG scores in the older people with sarcopenia (GS, WMD: 0.28 m/s; TUG, WMD: − 0.93 m/s). The results of subgroup analysis in our study showed that older than 70 years Asian people with sarcopenia had a significant increase in muscle performance after 12 weeks of resistance training.

In previous meta-analyses, specific recommendations have not been identified for resistance training prescriptions in older persons with sarcopenia [18, 19]. Nor are recommendations identified for the optimal frequency, duration, and intensity of resistance training for older people with sarcopenia. Therefore, we provide some recommendations for clinicians and practitioners who wish to prescribe resistance training in older populations with sarcopenia. According to the results of our subgroup analysis, resistance training should be kept at a moderate-high intensity (> 60% 1RM), two meta-analyses have also shown that high-intensity (> 70–75%1RM) resistance training is more effective in improving muscle strength and performance in older people than lower-intensity exercises [61, 62]. We also recommend a resistance training program of 3 days/week, with 2–3 sets of 8–12 repetitions for each movement. The mode of exercise should be appropriate to one’s abilities and interests. Older people should be able to choose the appropriate resistance training mode according to their needs and resources, such as elastic band and weight machines. We suggest that the older people should choose the elastic band as much as possible, because they are more likely to suffer injuries with weight machines than young people [63]. Regarding the duration of training (in weeks), longer duration are more effective than shorter duration in improving muscle strength. For example, in healthy older people, Borde et al. observed that 50–53 weeks of resistance training was more effective in increasing muscle strength than 6–9 weeks of resistance training [59]. Additional studies are needed to identify the optimal duration (in weeks) for resistance training to improve the effects of sarcopenia. It should be noted however, while resistance training can improve the effects of sarcopenia in older people, it cannot reduce the decline of age-related muscle strength. Thus, it is important that people perform resistance training throughout their lives, especially as they approach older age.

### Strengths and limitations

To the best of our knowledge, this is the first systematic review and meta-analysis aimed to assess the effects of resistance training on healthy older people with sarcopenia. The studies we included were high-quality randomized clinical trials. All the subjects in the studies had been diagnosed with sarcopenia according to identified criteria for sarcopenia. We excluded studies in which some of the subjects were not diagnosed for sarcopenia. In addition, all subjects in the intervention groups performed resistance training only, as we excluded studies that added aerobic training, balance training or nutritional supplementation. Our results were comprehensive to include changes in body composition, muscle strength, and muscle performance tests. The muscle strength and muscle performance tests reflected the effects of resistance training on the muscles of the upper and lower limbs. Our results showed that resistance training improves body fat mass, muscle strength and muscle performance and can be applied to the treatment and management of sarcopenia.

Our study also had some limitations. First, we included only 14 studies which might be due to our strict search and screening strategy. More RCTs are needed to have confidence in the positive benefits of resistance training for older people with sarcopenia in the future. Second, we included studies which aimed at obese older people with sarcopenia as little is known about how obesity effects the benefits of resistance exercise on sarcopenia. This may have led to differential effects on the resistance training responses in obese subjects as compared with leaner subjects. We conducted subgroup analyses and find that obese subjects have greater increases in HGS and KES than leaner subjects after resistance training. However, leaner subjects have greater gain in muscle mass from resistance training. More RCTs can be carried out in the future to explore the effect of fat mass on the benefit fro m exercise in older people with sarcopenia. Third, some of the results in our meta-analyses had high heterogeneity in terms of ASMI (*I*^*2*^ = 68%), HGS (*I*^*2*^ = 81%), KES (*I*^*2*^ = 67%) and GS(*I*^*2*^ = 68%), which might have been caused by different assessments and resistance training strategies in the studies included in the meta-analysis. The high heterogeneity likely indicated that there is still ambiguity in the evaluation and the resistance training prescriptions in the research studies. Thus, we conducted subgroup analyses to explore the influence of moderator variables (focus on participants features, resistance training modality) on muscle mass, HGS, KES, and muscle performance. Due to the limitations of the included articles, we have to integrate SMM, LLE and ASMI into muscle mass outcome, and integrate GS and TUG into muscle performance outcome. Forth, the intervention duration of the studies included in the meta-analysis were no longer than 36 weeks which may have limited changes in the muscle strength and performance effects observed in the meta-analysis. As noted earlier, more RCTs are needed to understand the long-term effects of resistance training in older people with sarcopenia.

## Conclusion

Our findings confirm the importance of resistance training in the treatment and management of sarcopenia in older people. Resistance training was able to improve the body fat mass, muscle strength and muscle performance. These findings will be strengthened by having additional high quality of RCTs of a longer duration to confirm the benefits of resistance training in older people with sarcopenia.

## Supplementary Information

**Additional file 1.** Search strategies in the systematic literature search.

## Data Availability

The datasets during and/or analysed during the current study available from the corresponding author on reasonable request.
