# Supplementary material for: Effects of resistance training in healthy older people with sarcopenia: a systematic review and meta-analysis of randomized controlled trials
Source: Eur Rev Aging Phys Act. 2021 Nov 11;18:23. doi: 10.1186/s11556-021-00277-7 (PMC8588688; doi:10.1186/s11556-021-00277-7)
Supplement: Supplementary file 1 — Additional file 1. Search strategies in the systematic literature search. [file 11556_2021_277_MOESM1_ESM.docx]

**Appendix**

**Search strategies**

1. **Search strategy for PubMed:**

#1 "Sarcopenia"[Mesh] OR "Muscular Atrophy"[Mesh] OR "Muscle Weakness"[Mesh] OR sarcopenia [Title/Abstract]OR sarcopenic[Title/Abstract] OR sarcopenie[Title/Abstract]

**#2** "Aged"[MeSH] OR "Elderly"[Title/Abstract] OR "Aging"[MeSH] OR "Senescence" [Title/Abstract] OR "biological aging"[Title/Abstract] OR "aging biological"[Title/Abstract] OR "aged, 80 and over" [MeSH Terms] OR "oldest old"[Title/Abstract] OR "Nonagenarians"[Title/Abstract] OR "Nonagenarian" [Title/Abstract] OR "Octogenarians"[Title/Abstract] OR "Octogenarian" [Title/ Abstract] OR "Centenarians" [Title/Abstract] OR "Centenarian"[Title/Abstract] OR "elderly frail" [Title/Abstract] OR "frail elders" [Title/Abstract]OR "elders"[All Fields] OR "Elders"[All Fields] OR "sambucus" [MeSH Terms] OR "sambucus" [All Fields] OR "Elder"[All Fields] OR "Frail" [Title/Abstract] OR "eldes"[All Fields] OR "Elders"[All Fields] OR "sambucus"[MeSH Terms] OR "sambucus"[All Fields] OR "Elder"[All Fields] OR "Frail"[Title/Abstract] OR "frail elder" [Title/Abstract] OR "functionally impaired elderly"[Title/Abstract] OR "elderly functionally impaired" [Title/Abstract] OR "functionally impaired elderly"[Title/Abstract] OR "frail older adults" [Title/Abstract] OR "Adult"[MeSH Terms] OR "Adult"[All Fields] OR "Adults"[All Fields] OR "adults"[All Fields] OR "frail older" [Title/Abstract] OR "adults frail older"[Title/Abstract] OR "frail older adult"[Title/Abstract] OR "older adult frail"[Title/Abstract] OR "older adults frail"[Title/Abstract] OR "Frail Elderly " [MeSH]

#3 "Resistance Training"[Mesh] OR "Weight Lifting"[Mesh] OR "Training, Resistance" [Title/Abstract] OR "Strength Training"[Title/Abstract] OR "Training, Strength"[Title/Abstract] OR"Weight-Lifting Strengthening Program"[Title/Abstract] OR "Strengthening Program, Weight-Lifting"[Title/Abstract] OR "Strengthening Programs, Weight-Lifting"[Title/Abstract] OR "Weight Lifting Strengthening Program"[Title/Abstract] OR "Weight-Lifting Strengthening Programs" [Title/ Abstract] OR "Weight-Lifting Exercise Program"[Title/Abstract] OR "Exercise Program, Weight-Lifting" [Title/Abstract] OR "Exercise Programs, Weight-Lifting" [Title/Abstract] OR "Weight Lifting Exercise Program"[Title/Abstract] OR "Weight-Lifting Exercise Programs"[Title/Abstract] OR "Weight-Bearing Strengthening Program"[Title/Abstract] OR "Strengthening Program, Weight-Bearing"[Title/Abstract] OR "Strengthening Programs, Weight-Bearing" [Title/Abstract] OR"Weight Bearing Strengthening Program"[Title/Abstract] OR "Weight-Bearing Strengthening Programs"[Title/Abstract] OR "Weight-Bearing Exercise Program"[Title/Abstract] OR "Exercise Program, Weight-Bearing" [Title/Abstract] OR "Exercise Programs, Weight-Bearing" [Title/Abstract] OR "Weight Bearing Exercise Program"[Title/Abstract] OR "Weight-Bearing Exercise Programs" [Title/Abstract] OR "Lifting, Weight [Title/Abstract]" OR "Liftings, Weight[Title/Abstract]" OR "Weight Liftings"[Title/Abstract]

#4 ("Single-Blind Method"[Mesh] OR "Double-Blind Method"[Mesh] OR "Randomized Controlled Trials as Topic"[Mesh] OR "Randomized Controlled Trial" [Publication Type] OR "Intention to Treat Analysis"[Mesh] OR "Controlled Clinical Trials as Topic"[Mesh] OR "Clinical Trials as Topic"[Mesh] OR "Clinical Trial" [Publication Type] OR "randomized controlled trial"[Publication Type] OR "random*"[Text Word] OR allocation[Text Word] OR "random allocation"[Text Word] OR" placebo [Text Word] OR" single blind"[Text Word] OR "double blind"[Text Word] OR "randomized controlled trial*"[Text Word] OR RCT[Text Word])

#5 #1 AND #2 AND #3 AND #4 204

1. **Search strategy for Cochrane Library:**

#1 "random*" or allocation or "random allocation" or placebo or single blind or double blind or "randomized controlled trial*" or RCT or "clinical trial*"#2 randomized controlled trial:pt or clinical trial:pt #3 'sarcopenia':ti,ab, #4 MeSH descriptor: [Sarcopenia] explode all trees, #5 MeSH descriptor: [Sarcopenia] explode all trees, #6 'aging':ti,ab or 'elderly':ti,ab or 'aged':ti,ab , #7 MeSH descriptor: [Aging] explode all trees, #8 MeSH descriptor: [Aged] explode all trees, #9 MeSH descriptor: [Frail Elderly] explode all trees, #10 MeSH descriptor: [Aged, 80 and over] explode all trees, #10(Exercise Programs, Weight-Lifting):ti,ab,kw OR (Strengthening Program, Weight-Lifting):ti,ab,kw OR (Weight-Lifting Strengthening Programs):ti,ab,kw OR (Weight-Lifting Strengthening Program) ti,ab,kw OR (Weight Lifting Strengthening Program):ti,ab,kw OR(Weight Lifting Exercise Program):ti,ab,kw OR (Strengthening Programs, Weight-Lifting):ti,ab,kw OR (Weight-Lifting Exercise Programs):ti,ab,kw OR (Weight-Lifting Exercise Program):ti,ab,kw OR (Exercise Program, Weight-Lifting):ti,ab,kw OR (Weight-Bearing Exercise Programs):ti,ab,kw OR (Weight-Bearing Strengthening Programs):ti,ab,kw OR (Exercise Program, Weight-Bearing):ti,ab,kw OR (Weight Bearing Strengthening Program):ti,ab,kw OR (Strengthening Programs, Weight-Bearing):ti,ab,kw OR (Weight-Bearing Exercise Program):ti,ab,kw OR (Exercise Programs, Weight-Bearing):ti,ab,kw OR (Weight-Bearing Strengthening Program):ti,ab,kw OR (Weight Bearing Exercise Program): ti, ab, kw OR (Strengthening Program, Weight-Bearing):ti,ab,kw OR(Training, Strength):ti,ab,kw OR (Training, Resistanc):ti,ab,kw OR (Strength Training):ti,ab,kw

#1 AND #2 AND (#3 OR #4 OR #5) AND (#6 OR #7 OR #8 OR #9) AND #10 2064

1. **Search strategy for Embase:**

#1 'randomization'/exp OR 'placebo'/exp OR 'placebo effect'/exp OR 'single blind procedure'/exp OR 'double blind procedure'/exp OR 'randomized controlled trial'/exp OR 'randomized controlled trial (topic)'/exp OR 'controlled clinical trial'/exp OR 'controlled clinical trial (topic)'/exp OR 'clinical trial'/exp OR 'clinical trial (topic)'/exp

#2 random*:ab,ti OR allocation:ab,ti OR "random allocation":ab,ti OR placebo:ab,ti OR single blind:ab,ti OR double blind:ab,ti OR randomised controlled trial*:ab,ti OR randomized controlled trial*:ab,ti OR RCT:ab,ti OR clinical trial*:ab,ti

#3 'sarcopenia':ab,ti OR 'sarcopenia'/exp AND ('aging':ab,ti OR 'elderly':ab,ti OR 'aged':ab,ti OR 'aging'/exp OR 'aged'/exp OR 'very elderly'/exp OR 'frail elderly'/exp)

#4'resistance training'/exp OR 'resistance exercise':ab,ti, OR 'resistance exercise training':ab,ti, OR 'strength training':ab,ti, OR 'weight bearing exercise':ab,ti

#5 (#1 OR #2) AND #3 AND #4 235

1. **Search strategy for China National Knowledge Infrastructure:**

（SU=随机 OR SU=随机分配 OR SU=随机对照 OR SU=对照 OR SU=盲法 OR SU=单盲 OR SU=双盲 OR SU=随机对照试验 OR SU=随机对照研究 OR SU=临床试验 OR SU=临床观察 OR SU=临床研究) AND (SU=老年人 OR SU=老年人，80 岁以上 OR SU=虚弱老人 OR SU=老人 OR SU=老年) AND (SU=抗阻训练 OR SU=抗阻运动 OR SU=对抗训练 OR SU=渐进式抗阻训练 OR SU=阻力运动 OR SU=力量运动 OR SU=弹力带训练) AND (SU=肌少症 OR SU=少肌症 OR SU=肌肉减少症 OR SU=骨骼肌减少症 OR SU=骨骼肌衰减症 OR SU=肌肉衰减综合征 OR SU=老年性骨骼肌衰减 OR SU=原发性老年肌肉衰减综合征) 28

1. **Search strategy for Wanfang Date:**

1# 随机 OR 随机分配 OR 随机对照 OR 对照 OR SU=盲法 OR 单盲 OR 双盲 OR 随机对照试验 OR 随机对照研究 OR 临床试验 OR 临床观察 OR 临床研究

2# 老年人 OR 老年人，80 岁以上 OR 虚弱老人 OR 老人 OR 老年

3# 抗阻训练 OR 抗阻运动 OR对抗训练 OR 渐进式抗阻训练 OR 阻力运动 OR 力量运动 OR弹力带训练

4# 肌少症 OR 少肌症 OR 肌肉减少症 OR 骨骼肌减少症 OR 骨骼肌衰减症 OR 肌肉衰减综合征 OR 老年性骨骼肌衰减 OR 原发性老年肌肉衰减综合征
